# Trends in Buprenorphine Coverage and Prior Authorization Requirements in US Commercial Formularies, 2017-2021

**DOI:** 10.1001/jamahealthforum.2022.1821

**Published:** 2022-07-08

**Authors:** Thuy D. Nguyen, Kao-Ping Chua, Barbara Andraka-Christou, W. David Bradford, Kosali Simon

**Affiliations:** 1 Department of Health Management and Policy, School of Public Health, University of Michigan, Ann Arbor; 2 Susan B. Meister Child Health Evaluation and Research Center, Department of Pediatrics, University of Michigan Medical School, Ann Arbor; 3 School of Global Health Management and Informatics, University of Central Florida, Orlando; 4 Department of Public Administration and Policy, University of Georgia, Athens; 5 O’Neil School of Public and Environmental Affairs, Indiana University, Bloomington; 6 National Bureau of Economic Research, Cambridge, Massachusetts

## Abstract

This cross-sectional study assesses buprenorphine coverage and prior authorization requirements in US commercial formulary data from 2017 to 2021.

## Introduction

Opioid overdose deaths in the US are at record levels. Buprenorphine, a medication used to treat opioid use disorder, decreases opioid-related mortality risk by 50%.^1^ Despite this, buprenorphine access is often impeded by insurance-related barriers.^2^ One study analyzed buprenorphine coverage and prior authorization requirements in the Health Insurance Marketplace private plans during 2017,^3^ but more recent data from a broader variety of commercial plans are lacking.

## Methods

In this cross-sectional study, we analyzed annual commercial formulary data from Ideon, a health information technology company that provides standardized plan design data from insurers to employee benefit managers and health technology companies. Because the database lacks patient information, the University of Michigan Medical School institutional review board exempted analyses from review. Informed consent was waived because data were deidentified. We followed the Strengthening the Reporting of Observational Studies in Epidemiology (STROBE) reporting guideline for cross-sectional studies.

The Ideon database includes formularies used by midsize and large group non-Marketplace plans, small group and individual Marketplace plans, and off-Marketplace small group and individual plans. Although Ideon lacks enrollment data for midsize and large group non-Marketplace plans, the small group and individual Marketplace plans associated with formularies in our sample collectively accounted for 8.6 million enrollees per year (eMethods 1 in the Supplement has additional details).^4^ For all individual and small group plans and most large group plans, Ideon collects information on network size, defined as the number of clinicians and facilities participating in the network.

We calculated the annual proportion of formularies covering 1 or more immediate-release buprenorphine product. Among these formularies, we calculated the proportion without prior authorization requirements for 1 or more immediate-release buprenorphine product. We conducted similar analyses for extended-release buprenorphine injection (Sublocade) and for individual products. We used Pearson χ^2^ test to compare proportions in 2017 and 2021.

In a sensitivity analysis, we weighted results by network size among formularies with network size data (eMethods 1 in the Supplement). We used Stata/MP, version 17.0 (StataCorp LLC) statistical software and 2-sided hypothesis tests with α = .05.

## Results

Analyses included 866 commercial formularies in the database throughout 2017 to 2021 (listed in eMethods 2 in the Supplement). Of the 866 formularies, 460 were associated with small group or individual Marketplace or off-Marketplace plans, and 406 were associated with large group plans.

From 2017 through 2021, the proportion of formularies covering specific immediate-release products changed, but the proportion covering 1 or more such product changed minimally, from 97.8% (847 of 866) in 2017 to 98.6% (854 of 866) in 2021 (*P* = .20). Among formularies covering 1 or more immediate-release buprenorphine product, the proportion without prior authorization requirements for 1 or more immediate-release buprenorphine product increased from 83.8% (710 of 847) in 2017 to 94.6% (808 of 854) in 2021 (*P* < .001), primarily owing to a considerable increase in 2018 (Figure). The proportion of formularies covering extended-release buprenorphine injection increased from 24.7% (214 of 866) in 2018, the first full year of product availability, to 46.2% (400 of 866) in 2021 (*P* < .001). Among these formularies, the proportion without prior authorization requirements for extended-release buprenorphine injection increased from 57.5% (123 of 214) in 2018 to 82.5% (330 of 400) in 2021 (*P* < .001) (Table).

**Figure.**
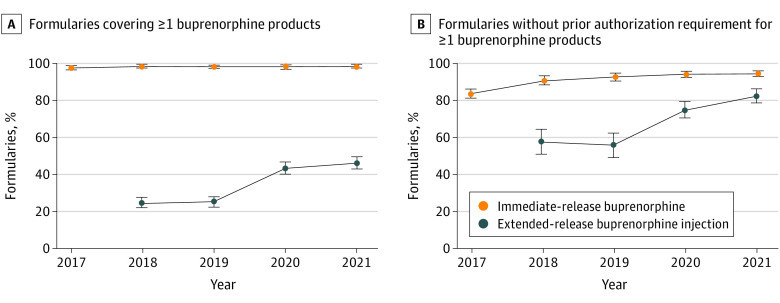
Coverage and Prior Authorization Requirements for Buprenorphine Products in Commercial Formularies, 2017-2021 A, Coverage of buprenorphine products in commercial formularies; B, Prior authorization requirements for buprenorphine products in commercial formularies. Data were derived from 866 commercial formularies included in the Ideon database throughout 2017 to 2021. The bars represent the 95% CIs for the proportions. The denominators for the proportions in Panel B are the numbers of formularies that covered at least 1 immediate-release buprenorphine product and the number that covered extended-release buprenorphine injection (Sublocade).

**Table.** Coverage and Prior Authorization Requirements for Buprenorphine Products in Commercial Formularies During 2017-2021

	Main analysis: 866 formularies included in the database, 2017-2021			Sensitivity analysis: 451 formularies with network size data, results weighted by No. of unique NPIs^a^		
	No. (%)		*P* value^b^	No. (%)		*P* value^b^
	2017	2021		2017	2021	
**Immediate-release buprenorphine: % formularies covering the product**						
≥1 Immediate-release buprenorphine product	847 (97.8)	854 (98.6)	.20	450 (99.8)	450 (99.8)	>.99
≥1 Immediate-release film	791 (91.3)	820 (94.7)	.006	411 (91.1)	426 (94.5)	.053
≥1 Immediate-release tablet	810 (93.5)	840 (97.0)	<.001	447 (99.1)	449 (99.6)	.41
Suboxone (sublingual buprenorphine/naloxone film and tablet)	810 (93.5)	738 (85.2)	<.001	426 (94.5)	375 (83.1)	<.001
Generic buprenorphine/naloxone film^c^	471 (54.4)	566 (65.4)	<.001	415 (88.1)	527 (93.1)	.005
Bunavail (buccal buprenorphine/naloxone film)	583 (67.3)	612 (70.7)	.13	311 (69.0)	322 (71.4)	.42
Zubsolv (buprenorphine/naloxone tablet)	639 (73.8)	717 (82.8)	<.001	345 (76.5)	374 (82.9)	.016
Generic buprenorphine/naloxone tablet	771 (89.0)	774 (89.4)	.82	250 (55.4)	316 (70.1)	<.001
Generic buprenorphine tablet	774 (89.4)	792 (91.5)	.14	432 (95.8)	440 (97.6)	.14
**Immediate-release buprenorphine: among formularies covering the product, % without prior authorization requirements**						
≥1 Immediate-release buprenorphine product	710 (83.8)	808 (94.6)	<.001	347 (77.1)	423 (94.0)	<.001
≥1 Immediate-release film	471 (59.5)	707 (86.2)	<.001	205 (49.9)	358 (84.0)	<.001
≥1 Immediate-release tablet	667 (82.3)	790 (94.0)	<.001	339 (75.8)	418 (93.1)	<.001
Suboxone (sublingual buprenorphine/naloxone film and tablet)	622 (76.8)	628 (85.1)	<.001	294 (69.0)	307 (81.9)	<.001
Generic buprenorphine/naloxone film^c^	415 (88.1)	527 (93.1)	.005	207 (82.8)	290 (91.8)	.001
Bunavail (buccal buprenorphine/naloxone film)	315 (54.0)	415 (67.8)	<.001	145 (46.6)	194 (60.2)	<.001
Zubsolv (buprenorphine/naloxone tablet)	467 (73.1)	559 (78.0)	.037	228 (66.1)	274 (73.3)	.036
Generic buprenorphine/naloxone tablet	471 (61.1)	637 (82.3)	<.001	226 (53.1)	346 (82.0)	<.001
Generic buprenorphine tablet	411 (53.1)	600 (75.8)	<.001	205 (47.5)	345 (78.4)	<.001
**Extended-release buprenorphine** ^d^						
% Formularies covering extended-release buprenorphine injection	214 (24.7)	400 (46.2)	<.001	115 (25.5)	220 (48.8)	<.001
Among formularies covering extended-release buprenorphine injection, % without prior authorization requirements	123 (57.5)	330 (82.5)	<.001	54 (47.0)	175 (79.5)	<.001

Abbreviation: NPI, National Provider Identifier.

^a^ Of the 866 formularies included in the database, 451 (52.1%) contained data on the number of unique NPIs among clinicians and institutions that were in-network for the plans using the formulary.

^b^ Pearson χ^2^ test was used to assess differences in proportions between 2017 and 2021.

^c^ Data from 2019 to 2021 were used for generic buprenorphine/naloxone film. The first of such products was approved in June 2018 by the US Food and Drug Administration. Although entry was delayed by a patent infringement lawsuit from the manufacturer of the branded version (Suboxone), the generic product eventually was allowed to launch in February 2019.

^d^ Data from 2018 to 2021 were used for extended-release buprenorphine injection (Sublocade) because the drug was approved in December 2017, meaning that 2018 was the first full year in which this drug was available. We did not assess extended-release buprenorphine implant (Probuphine), as this product was discontinued in 2020.

A total of 451 formularies had network size data. For these formularies, weighting by network size did not change conclusions (Table).

## Discussion

In this cross-sectional study sample of commercial formularies used by large group, small group, and Marketplace plans, almost all covered 1 or more immediate-release buprenorphine products from 2017 through 2021. An increasing share did so without requiring prior authorization. In 2021, only 5.4% of these formularies required prior authorization for all covered immediate-release products. In contrast, among formularies covering extended-release buprenorphine injection in 2021, 17.5% required prior authorization, even though extended-release buprenorphine products can improve adherence and patient satisfaction.^5^

Limitations include unclear generalizability of results to all commercial formularies. Findings would underestimate extended-release buprenorphine injection coverage if plans were covering this drug as a medical benefit and if the formulary database only were capturing drugs covered under the pharmacy benefit. In addition, the database did not report details of prior authorization requirements.

Nevertheless, these findings highlight potential opportunities to improve commercial insurance benefit design for buprenorphine, particularly extended-release buprenorphine injection. In light of record opioid-related mortality, private plans should strongly consider following the approach of Medicare Part D plans, which almost completely eliminated prior authorization requirements for buprenorphine products in 2019.^6^

## Notes

Supplement. **eMethods 1. **Additional details on the Ideon formulary database and the study sample **eMethods 2. **Names of formularies included in the sample
