# Supplementary material for: Trends in Buprenorphine Coverage and Prior Authorization Requirements in US Commercial Formularies, 2017-2021
Source: JAMA Health Forum. 2022 Jul 8;3(7):e221821. doi: 10.1001/jamahealthforum.2022.1821 (PMC9270692; doi:10.1001/jamahealthforum.2022.1821)
Supplement: Supplement. — eMethods 1. Additional details on the Ideon formulary database and the study sample eMethods 2. Names of formularies included in the sample [file jamahealthforum-e221821-s001.pdf]

Supplemental Online Content

Nguyen TD, Chua KP, Andraka-Christou B, Bradford WD, Simon K. Trends in buprenorphine coverage and prior authorization requirements in US commercial formularies, 2017-2021. *JAMA Health Forum.* 2022;3(7):e221821. doi:10.1001/jamahealthforum.2022.1821

eMethods 1. Additional details on the Ideon formulary database and the study sample

eMethods 2. Names of formularies included in the sample

This supplemental material has been provided by the authors to give readers additional information about their work.

© 2022 Nguyen TD et al. *JAMA Health Forum.*

eMethods 1. Additional details on the Ideon formulary database and the study sample

Description of Ideon

Ideon is a health information technology company that works directly with health insurers, companies that manages benefits for employees, and technology companies. Health insurance plans directly provide data on their medical and pharmacy benefit designs and provider networks to Ideon. Additionally, Ideon utilizes webscrapes of online plan directories to augment the provider network data. Ideon then converts these data into a standardized format. The standardized data helps employee benefit managers and employees understand differences in plan design when selecting plans. Moreover, Ideon's standardized data are used by companies that offer platforms to help patients find doctors that are in a particular provider's network. Ideon is responsible for the Health Insurance Exchange (HIX) Compare data,[1] a publicly available database with plan-level information on all every individual and small group plan offered in the Marketplace in all 50 states and the District of Columbia, as well as most off-Marketplace individual and small group plans.

Ideon formulary data

This study analyzed formularies for private plans in Ideon's formulary database.[2] This database contains formularies used in individual plans, small group plans, and mid to large group plans. The formulary data provides national drug code-level detail on which drugs are covered by which plans and at what tier level, along with whether any restrictions such as prior authorization are imposed. Ideon's formulary data are comprehensive. For example, the 2021 formulary data

[1] https://hixcompare.org/
[2] https://ideonapi.com/researchers/

© 2022 Nguyen TD et al. *JAMA Health Forum.*

contain 419,288,021 records at the formulary-NDC level of 168,146 NDCs, representing approximately 80% of all NDCs listed by the FDA.[3] The formulary database has been used in prior studies.[4]

Sample details

HIOS IDs are a plan identifier assigned by CMS. These identifiers uniquely identify CMS-approved qualified health plans (individual or small group) that can be sold on the Marketplace, as well as individual or small group plans that are sold off the Marketplace. The CMS Center for Consumer Information and Insurance Oversight (CCIIO) provides one plan-level database that contains HIOS IDs and plan name of Marketplace plans (Plan Finder list), and another database that contains HIOS ID and number of plan enrollees in Marketplace plans (Issuer Level Enrollment data).

Our sample of 866 commercial formularies included 406 that did not have a HIOS ID, signifying that they were mid-large group plans. Unfortunately, Ideon does not have data on enrollment in these plans. A total of 460 formularies did have HIOS IDs, signifying that they were individual or small group Marketplace or off-Marketplace plans. Of these 460 formularies, 437 had HIOS IDs that mapped to a plan in the CCIIO Plan Finder list. These 437 formularies were associated with a total of 4,034 plans. These plans collectively accounted for 8.6 million Marketplace enrollees on average during 2017-2020, representing 96.4% of all 8.9 federally-facilitated Marketplace enrollees.

---

[3] https://www.fda.gov/drugs/drug-approvals-and-databases/national-drug-code-directory, accessed on April 9, 2022.
[4] McManus KA, Powers S, Killelea A, Tello-Trillo S, McQuade ER. Regional disparities in qualified health plans' prior authorization requirements for HIV pre-exposure prophylaxis in the United States. JAMA Network Open. 2020 Jun 1;3(6):e207445-.

Beilfuss S, Linde S. Pharmaceutical opioid marketing and physician prescribing behavior. Health Economics. 2021 Dec;30(12):3159-85.

© 2022 Nguyen TD et al. *JAMA Health Forum.*

Among the 866 formularies, 451 had information on network size. The number of unique NPIs across plans associated with these formularies was 2.4-2.5 million during 2017-2021, representing 33.7-48% of all NPIs in the NPI Registry Database.

© 2022 Nguyen TD et al. *JAMA Health Forum.*

eMethods 2. Names of formularies included in the sample

To provide readers with context on the nature of our sample, the full list of 866 formularies in this study is listed below.

4-D Management Systems
AFSCME Council 31
ASR Corporation Employees
ATRIO Marketplace HIX OR
Advanced Control Formulary for Teachers Health Trust
Adventist Health
Aetna 5 Tier HIX
Aetna Commercial Fully Insured 2 Tier Closed
Aetna Commercial Fully Insured 2 Tier Open
Aetna Commercial Fully Insured 3 Tier
Aetna Commercial Fully Insured 4 Tier
Aetna Commercial Fully Insured 5 Tier
Aetna Commercial Self Insured 2 Tier Closed
Aetna Commercial Self Insured 2 Tier Open
Aetna Commercial Self Insured 3 Tier
Aetna Commercial Self Insured 4 Tier
Aetna Commercial Self Insured 5 Tier
Aetna HIX Florida
Aetna Innovation Health Leap HIX
Aetna Leap HIX
Aetna Premier 2 Tier
Aetna Premier 3 Tier
Aetna Premier 4 Tier
Aetna Premier 5 Tier
Aetna Premier Plus 2 Tier
Aetna Premier Plus 3 Tier
Aetna Premier Plus 4 Tier
Aetna Premier Plus 5 Tier
Aetna Value 2 Tier Closed
Aetna Value 2 Tier Open
Aetna Value 3 Tier
Aetna Value 4 Tier
Aetna Value 5 Tier
Aetna Value Plus 2 Tier
Aetna Value Plus 3 Tier
Aetna Value Plus 4 Tier
Aetna Value Plus 5 Tier
Aetna Value Plus Small Group 3 Tier
Aetna Value Plus Small Group 3 Tier NJ
Aetna Value Plus Small Group 4 Tier
Aetna Value Plus Small Group 4 Tier MD and WV
Aetna Value Plus Small Group 5 Tier
Aetna Value Plus Small Group 5 Tier DE
Aetna Value Plus Small Group 5 Tier FL
Aetna Value Small Group 5 Tier
Affinity Essential Qualified Health Plan
Allegian Choice HIX
Allegian Health Plans 4-Tier Closed
Alliant Energy 2 Tier
Alliant Energy 3 Tier
Alliant Health Plans 3 Tier Formulary
Alliant Health Plans Essential Health Benefits Formulary
Alliant Plan Plus/Select 2 Tier
Ambetter IlliniCare HIX
Ambetter NH HIX
Ambetter from Buckeye Community Health Plan HIX OH
Ambetter from CeltiCare Health Plan
Ambetter from Coordinated Care HIX
Ambetter from MHS Health Wisconsin HIX
Ambetter from MHS Indiana HIX
Ambetter from Magnolia Health Plan
Ambetter from Peach State Health Plan HIX
Ambetter from Sunshine Health HIX
Ambetter from Superior Health Plan HIX
Ambetter of Arkansas HIX
Ameriben Employees
American Casino and Entertainment Properties
American Health Care
American Health Care Garden Grove Unified School District
American Health Care Pinnacle
Anthem BC GenRX
Anthem BCBS HIX NV
Anthem Blue Cross Generic Premium
Anthem BlueCross BlueShield HIX CO
Anthem BlueCross BlueShield HIX CT
Anthem BlueCross BlueShield HIX IN
Anthem BlueCross BlueShield HIX KY
Anthem BlueCross BlueShield HIX ME
Anthem BlueCross BlueShield HIX MO
Anthem BlueCross BlueShield HIX NH
Anthem BlueCross BlueShield HIX OH
Anthem BlueCross BlueShield HIX VA
Anthem BlueCross BlueShield HIX WI
Anthem BlueCross HIX CA
Anthem COVA Care
Anthem Four Tier
Anthem National Closed
Anthem Three Tier
Appvion Formulary
Arise Health Plan
Arizona Department of Health Services (ADHS)
Arizona FCU
Arizona Local Government Employee Benefit Trust
Arizona Metropolitan Trust
Arkansas BlueCross BlueShield Formulary One (Standard)
Arkansas BlueCross BlueShield HIX

© 2022 Nguyen TD et al. *JAMA Health Forum.*

Arkansas State and Public School Employees
AultCare
AultCare MarketPlace HIX OH
AultCare MarketPlace HIX OH Closed
Auto Wares Group
AvMed Engage and Empower Formulary
AvMed Health Plans
AvMed Miami Dade County HMO Medication Formulary
Avera Health Plan HIX
Avera Health Plans
BC Northeastern PA
BC OF IDAHO 2 TIER
BC OF IDAHO 3 Tier
BC of Idaho Qualified Health Plans
BCBS Alabama
BCBS FEP Basic
BCBS FEP Standard
BCBS IL, MT, NM, OK, TX Generics Plus 4 Tier
BCBS Illinois HMO
BCBS Kansas City KS Blue & U HIX
BCBS Kansas City MO 2 Tier
BCBS Kansas City MO 3 Tier
BCBS Kansas City MO Blue & U HIX
BCBS MI 3 Tier Clinical Formulary
BCBS MI Preferred Rx 2T Closed
BCBS Massachusetts 3 Tier HIX
BCBS Massachusetts Three Tier
BCBS Massachusetts Two Tier
BCBS Michigan 5 Tier HIX
BCBS Mississippi 4 TIER
BCBS Mississippi Preferred PPO
BCBS Montana 3 Tier
BCBS Tennessee HIX
BCBS Tennessee Standard Formulary
BCBS of AZ HIX EverydayHealth, TrueHealth and Essential
BCBS of Alabama 4 Tier
BCBS of Alabama Generics Plus 3 Tier
BCBS of Alabama Generics Plus 4 Tier
BCBS of Alabama Generics Plus Closed
BCBS of Alabama PrimeChoice Essential HIX
BCBS of Alabama Standard HIX
BCBS of Alabama Tiered Generic
BCBS of Arizona HIX Portfolio & SimpleHealth
BCBS of Arizona with Specialty
BCBS of Georgia HIX GA
BCBS of Illinois
BCBS of Illinois Marketplace 5 Tier Generics Plus HIX
BCBS of Kansas BlueCare HIX
BCBS of Kansas National/Select Three Tier
BCBS of Kansas National/Select Two Tier Closed
BCBS of Kansas National/Select Two Tier Open
BCBS of Louisiana 2 Tier
BCBS of Louisiana 4 Tier
BCBS of MI 2 Tier Clinical
BCBS of Michigan Preferred Rx 2 Tier Open
BCBS of Michigan Preferred Rx Three Tier
BCBS of Minnesota FlexRx 2 Tier
BCBS of Minnesota FlexRx Closed
BCBS of Minnesota FlexRx Three Tier
BCBS of Minnesota GenRx 4 Tier
BCBS of Minnesota GenRx MN HIX
BCBS of Montana EfficientRx/Generics Plus
BCBS of Montana Marketplace Generics Plus HIX
BCBS of Nebraska Individual 5 Tier HIX
BCBS of New Mexico
BCBS of New Mexico 4 Tier
BCBS of North Carolina Basic 5T Closed
BCBS of North Carolina Basic 5T Open
BCBS of North Carolina Basic 5T Open HIX
BCBS of North Carolina Enhanced 4 Tier
BCBS of North Carolina Enhanced 5 Tier
BCBS of North Dakota
BCBS of North Dakota HIX
BCBS of Oklahoma 4 Tier
BCBS of Oklahoma Marketplace 5 Tier Generics Plus
BCBS of South Carolina Blue Option HIX
BCBS of South Carolina BlueEssentials HIX
BCBS of South Carolina Preferred
BCBS of South Carolina Preferred 3 Tier with Specialty
BCBS of South Carolina Try Generics
BCBS of Texas 3 Tier
BCBS of Texas 4 Tier
BCBS of Texas HIX 5 Tier Generics Plus
BCBS of Vermont
BCBS of Western NY Formulary 1
BCBS of Western NY Formulary 2
BCBS of Western NY Formulary 3/Exclusive Small Group
BCBS of Wyoming
BCBS of Wyoming BlueSelect 3 Tier HIX
BCBS of Wyoming BlueSelect 4 Tier HIX
BCN MI Blue Essentials
BCN MI Custom 5T Formulary
BCN MI Custom Formulary Three Tier
BCN Michigan Custom Formulary 2 Tier Closed
BCN Michigan Custom Formulary 2 Tier Open
BCN Michigan HIX MI
BMC HealthNet ConnectorCare/Qualified Health Plan
Baptist Health Plan KY HIX
Baptist Health Plan Master Preferred Four Tier
Baptist Health Plan Master Preferred Three Tier
BayCare Health System
BeneCard PBF
Black Hawk County 1 Formulary
Black Hawk County 2 Formulary
Black Hawk County 3 Formulary

© 2022 Nguyen TD et al. *JAMA Health Forum.*

Blue Care Network of Michigan FEHBP
Blue Cross Blue Shield LA 3 Tier
Blue Cross Blue Shield Nebraska Standard 4 Tier
Blue Cross Blue Shield of Nebraska
Blue Cross Blue Shield of Rhode Island 5 Tier
Blue Cross Blue Shield of Rhode Island HIX
Blue Cross Blue Shield of Rhode Island Large Group 4 Tier
Blue Cross Blue Shield of Rhode Island Large Group 5 Tier
Blue Cross Blue Shield of Western NY HIX
Blue Selections Louisiana 5 Tier
Blue Shield HIX California
Blue Shield of California Plus Formulary
Blue Shield of Northeastern NY HIX
BlueChoice HealthPlan of South Carolina Tiered
BlueChoice/BlueChoice Plus
BlueCross BlueShield of Georgia 3 Tier
BlueCross BlueShield of Georgia 4 Tier
BlueCross BlueShield of Oklahoma/ BlueLincs
BlueShield of Northeastern NY Formulary 2
BlueShield of Northeastern NY Formulary 3
BlueShield of Northeastern New York Formulary 1
Board of Pensions of the Presbyterian Church
Board of Regents of The University System of Georgia
Bridgespan Essential HIX ID
Bridgespan Essential HIX OR
Bridgespan Essential HIX WA
Bridgespan Standard Formulary HIX OR
CDPHP Formulary 2 HIX NY
CHG Staffing
CHRISTUS Health Plan New Mexico HIX
CHRISTUS Health Plan Texas HIX
CVS Caremark Advanced Control Formulary
CVS Caremark Performance Formulary
CVS Caremark Performance w/ Advanced Specialty Control
CVS Caremark Performance w/ Exclusions
CVS Caremark Performance w/ Exclusions & Advanced Specialty Control
CVS Caremark Value
CalPERS
Capital Blue Cross 4 Tier
Capital BlueCross
Capital BlueCross Closed
Capital BlueCross HIX PA
Capital Health Plan FEHBP
CareFirst Formulary 2 3 Tier
CareFirst Formulary 2 4 Tier
CareFirst Formulary 2 HIX
CareSource Just4Me HIX IN
CareSource Just4Me HIX KY
CareSource Just4Me HIX OH
CareSource Just4Me HIX WV
Cascade Die Casting
Caterpillar Drug Formulary
Central PA Teamsters
Chinese Community Health Plan Commercial
Chinese Community Health Plan HIX
Cigna Generics Only
Cigna Legacy Three Tier
Cigna Performance Four Tier
Cigna Performance Three Tier
Cigna Rx Essential 5-Tier HIX
Cigna Rx Plus HIX TX, GA, and TN
Cigna Rx Premier HIX
Cigna Standard Three Tier
Cigna Value Three Tier with DRT
Cigna Value Three Tier without DRT
City of Austin Traditional 3 Tier
City of Janesville 2 Tier
City of Janesville 3 Tier
City of La Crosse
City of North Platte
Clear Script Formulary
Cleveland Clinic EHP Drug Formulary
Commercial Roofing Benefits
Common Ground Healthcare Cooperative HIX WI
Community Bank and Trust
Community First Health Plans
Community First Health Plans HIX TX
Community Health Choice HIX TX
Community Health Plan of Washington Apple Health
Community Health Plan of Washington HIX
ConnectiCare 3 Tier
ConnectiCare Freedom HIX CT
CoventryOne FL HIX
CoventryOne HIX
Cox Health Plan
Cox Smith Matthews Incorporated
Crystal Run Health Commercial
Culinary Health Fund Formulary
Cummins Custom
Cypress Care Workers' Compensation Drug List
DTR Industries
DakotaCare
Dalton Corporation
Dean Health Plan HIX WI
Dean Health Plan Three Tier
Dean Health Plan Two Tier
Denver Health Elevate HIX CO
Doctors Hospital at Renaissance
ELCA Board of Pensions
EMI Health Educators Rx Basic
East Liverpool City Hospital
Eli Lilly Drug List
EmblemHealth Select Care HIX
Empire BCBS Select HIX NY
Empire BlueCross HIX NY

© 2022 Nguyen TD et al. *JAMA Health Forum.*

Envolve
Evergreen Health Cooperative HIX MD
Excellus BCBS 3 Tier
ExcellusBCBS HIX NY
Excelsior NY State Employees Plan
Exclusive Care
Express Scripts Basic Formulary
Express Scripts Basic with Limited
Express Scripts EGWP High Performance 3 Tier
Express Scripts EGWP National Preferred 3 Tier
Express Scripts EGWP National Preferred 4 Tier
Express Scripts High Performance
Express Scripts High Performance with Limited
Express Scripts National Preferred
Express Scripts National Preferred with Limited
Fallon Community Health Plan
Fallon Community Health Plan HIX MA
Fidelis Care Exchange NY
FirstCare Health Plans Commercial 5 Tier Formulary
FirstCare Health Plans Marketplace Formulary (HIX TX)
Florida Blue Care Choices HIX
Florida Blue Three Tier
Florida Blue Two Tier Closed
Florida Blue ValueScript Rx HIX
Florida Health Care Plan HIX
GHI City of New York Employee Plans
GL Noble Denton Formulary
Geisinger Health Plan Traditional
Geisinger Health Plan Triple Choice
Geisinger Marketplace Formulary
General Prescription Programs
Genesis, Genesis Prime, Genesis at Home, and Orion Constellation Health
Georgia State Health Benefit Plan
Gill Industries Formulary
GlobalHealth FEHBP
GlobalHealth State Employee & Educator Plan
Government Employee Health Association (GEHA)
Group Health Cooperative HIX WA
Group Health Cooperative South Central Wisconsin HIX
Group Health Cooperative Washington
Group Health Cooperative of S Central WI Complete Three Tier
Gundersen Lutheran Health Plan
HIP Health Plan New York 2 Tier
HMO Louisiana 3 Tier
HMO Partners, Inc.
HMSA Choice
HMSA Choice Non-Grandfathered Plans
HMSA Control
HMSA Essential
HMSA Metallic Prescription Formulary
HMSA Select
HMSA Select Non-Grandfathered Plans
Harbor Health Plan HIX
Harken Health GA HIX
Harken Health IL HIX
Harris Corporation
Harvard Pilgrim Premium 3 Tier
Harvard Pilgrim Premium 4 Tier
Harvard Pilgrim Stride and Stride of NH
Harvard Pilgrim Value Formulary 3 Tier
Harvard Pilgrim Value Formulary 4 Tier
Harvard Pilgrim Value Formulary 5 Tier
Health Alliance Medical Plans HIX
Health Alliance Plan HIX MI
Health Alliance Plan of Michigan 2-Tier
Health Alliance Plan of Michigan 3-Tier
Health Choice Arizona Essential HIX AZ
Health First Health Plans Florida 3Tier
Health First Health Plans Florida 5 Tier
Health First Health Plans Florida HIX
Health Net Washington Essential Rx
Health Net of AZ 3 Tier Standard with Specialty
Health Net of Arizona 3 Tier Simplified with Specialty
Health Net of Arizona 4 Tier Simplified with Specialty
Health Net of Arizona 4 Tier Standard with Specialty
Health Net of Arizona Essential Rx HIX
Health Net of California Essential Rx HIX
Health Net of California Three Tier
Health Net of California Three Tier with Specialty
Health Net of California Two Tier with Specialty
Health Net of Oregon Essential Rx
Health New England
Health Plan of Nevada 4 Tier HIX
Health Plan of Nevada Three Tier
Health Plans of Nevada Four Tier
Health Republic New Jersey HIX
Health Select of Texas
Health Tradition
Health Tradition HIX
HealthAmerica Pennsylvania B1 Formulary
HealthAmerica Pennsylvania B2 Formulary
HealthAmerica Pennsylvania B2 Formulary Closed
HealthAmerica Pennsylvania B3 Formulary
HealthLinc
HealthNow New York Three Tier
HealthPartners MN HIX GenericsAdvantage 2 Tier
HealthPartners MN HIX GenericsAdvantage 4 Tier
HealthPartners Minnesota
HealthPartners Minnesota FEHBP
HealthPartners Minnesota Health Care Programs
HealthPartners UnityPoint Health-Align-Symmetry
HealthPlus County Health Plan A and B
HealthPlus Enterprise & Large Group Commercial
HealthPlus HMO/PPO Legacy Commercial
HealthPlus VenturePlus & Signature Commercial

© 2022 Nguyen TD et al. *JAMA Health Forum.*

HealthSmartRx
HealthSpan HIX OH
HealthSpan Selected Formulary
HealthSpan Standard Formulary 3 Tier
HealthSpan Standard Formulary 4 Tier
HealthSpan Traditional Formulary 2 Tier
HealthSpan Traditional Formulary 3 Tier
Healthfirst Leaf Plans HIX NY
HealthyCT HIX
Hennepin County
Highmark BCBS Delaware
Highmark BCBS Delaware HIX Comprehensive
Highmark BCBS HIX Comprehensive
Highmark BCBS HIX Progressive
Highmark Blue Cross Blue Shield 3 Tier
Highmark Health Options
Highmark Progressive Formulary
Hillsborough County Public Schools Rx4
Home State Health Plan
Hometown Standard
Horizon BCBS of New Jersey
Horizon BCBS of New Jersey Two Tier
Horizon BlueCross BlueShield of NJ HIX
Horizon NJ Health
Hospitality Rx Formulary
Humana HDHP Complete HIX FL
Humana HDHP Complete HIX TX
Humana HDHP EHB HIX
Humana HDHP Plus HIX
Humana HDHP Traditional
Humana HDHP Value HIX CO and UT
Humana National 5 Closed
Humana National 5 Formulary
Humana National 5 PDP Enhanced
Humana National 5 w/ ED Drugs
Humana National 5 w/ Home Infusion
Humana National 5 w/ Maintenance Medications
Humana National 5 with GC
Humana National 5 with Weight Loss
Humana National 6 - Diabetes
Humana National 6 CSNP
Humana National 6 w/ ED & Maintenance Medications
Humana National 6 w/ Maintenance Medications
Humana National 6 with Home Infusion
Humana Preferred Rx
Humana Rx2 EHB
Humana Rx2 Traditional
Humana Rx3 EHB
Humana Rx3 Standard
Humana Rx3 Traditional
Humana Rx4 EHB
Humana Rx4 EHB HIX
Humana Rx4 Standard
Humana Rx4 Traditional
Humana Rx5 Complete HIX FL
Humana Rx5 Complete HIX TX
Humana Rx5 Plus HIX
Humana Rx5 Value HIX CO and UT
Humana RxImpact
Humana Walmart Rx Plan
IBM
INTotal Health
IU Health Plans HIX
Iberia Medical Center
IdealCare from Sendero Health Plans
InHealth Mutual HIX
Independence BC AmeriHealth Select
Independence Blue Cross HIX 4 Tier
Independence Blue Cross HIX 5 Tier
Independence Blue Cross/AmeriHealth NJ HIX 3 Tier
Independent Health EGWP
Independent Health FEHBP
Independent Health HIX NY
Independent Health-Western New York
Indiana University Health Plan
Integrated Prescription Management Basic Plus
Intel Providence Health Plan
International Foundation of Employee Benefit Plans
Irwin Telescopic Seating
Jai Medical Systems
Johns Hopkins EHP Formulary
Johnsonville Sausage
KPS Health Plans
Kaiser Permanente Colorado - Denver/Boulder/Northern HIX
Kaiser Permanente Colorado - Southern HIX
Kaiser Permanente Colorado PPO
Kaiser Permanente Denver/Boulder, Mountain and Northern Colorado HMO
Kaiser Permanente EPO & Self Funded Colorado
Kaiser Permanente FEHBP Colorado
Kaiser Permanente Georgia HIX
Kaiser Permanente Georgia HMO
Kaiser Permanente Georgia Multi-Choice
Kaiser Permanente Hawaii
Kaiser Permanente Hawaii HIX 3 Tier
Kaiser Permanente Hawaii HIX 4 Tier
Kaiser Permanente Hawaii QUEST
Kaiser Permanente Maryland HealthChoice
Kaiser Permanente Mid-Atlantic
Kaiser Permanente Mid-Atlantic HIX
Kaiser Permanente Northern California
Kaiser Permanente Northern California HIX
Kaiser Permanente Northwest
Kaiser Permanente Oregon HIX
Kaiser Permanente S. Colorado HMO 2 Tier
Kaiser Permanente Southern California

© 2022 Nguyen TD et al. *JAMA Health Forum.*

Kaiser Permanente Southern California HIX
Kaiser Permanente Southern Colorado HMO Formulary
Kaiser Permanente Washington HIX
Kalamazoo Anesthesiology
Kansas State Group Health Insurance Program
Kentucky Employees Health Plan 3 Tier
Kentucky Rural Electric Cooperative
Kroger Pharmacy Generic Formulary
Kroger Prescription Plans
LA Care Marketplace HIX CA
LDI Pharmacy Benefit Management
Land of Lincoln HIX IL
MAPFRE Formulary
MDWise HIX
MODA Health Plan
MVP Health Care HIX NY
MVP Health Care HIX VT
MVP Health Plan
MVP Health Plan Formulary
MVP Option and Option Family
Magellan Rx Precision
Magellan Rx Standard Formulary
Magellan Rx Standard Plus
Magic Steel Corporation
Mail Handlers Benefit Plan
Maine Community Health Options
Maryland Physicians Care
MaxCare
Maxor Plus Three Tier
Maxor Plus Two Tier
Mayo Clinic 2 Tier
Mayo Clinic 3 Tier
Mayo Health Employee Formulary
McLaren Health Plan Commercial
McLaren Health Plan HIX MI
MedImpact Portfolio
MedImpact Select
MedTrak National
Medica Advanced Control Specialty
Medica HIX
Medica SelectCare Two Tier
Medica Two Tier
Medical Associates Health Plan
Medical Mutual of Ohio
Medical Mutual of Ohio HIX
MercyCare Health Plans HIX WI
Meridian Choice HIX MI
Metalcraft of Mayville
MetroPlus Health Plan HIX NY
Minuteman Health HIX MA
Mississippi State and School Employees Health Plan
Moda Health Large Groups
Moda Health Plan Small Group Formulary
Moda Health Plan of Oregon HIX
Moda Health Preferred Drug List
Moda Health Preferred Drug List of Alaska HIX
Molina Healthcare of California HIX
Molina Healthcare of Florida HIX
Molina Healthcare of Michigan HIX
Molina Healthcare of New Mexico HIX
Molina Healthcare of Ohio HIX
Molina Healthcare of Texas HIX
Molina Healthcare of Utah HIX
Molina Healthcare of Washington HIX
Molina Healthcare of Wisconsin HIX
Montana Health Coop HIX MT
Montana State Fund
Montgomery County Public Schools
Mountain Health Coop HIX
MyPriority MI 5 Tier HIX
MyPriority MI 6 Tier HIX
Nashville Metro Government
National Benefit & Pension Fund 1199
National Pharmaceutical Services/Pharmaceutical Technologies, Inc.
Navitus Open Formulary
Navitus Select
Navitus Traditional Formulary
Neighborhood Health Plan MA HIX
Neighborhood Health Plan of RI HIX
Network Health Plan WI HIX
New Mexico Health Connections HIX NM
New York Hotel Trades Council/Hotel Association of New York City
Nippon Life
North Carolina State Health Plan for Teachers and State Employees
North Shore LIJ CareConnect HIX NY
Northwest Pharmacy Services
Ohio Bureau of Workers' Compensation
Ohio Public Employees Retirement System (OPERS) Non-Medicare
Optima Health Four Tier
Optima Health Plan HIX VA
OptumRx Generic-Centric
OptumRx Premium Highly Managed
OptumRx Premium Highly Managed with UM
OptumRx Select Covered
OptumRx Select Covered with Focused UM
OptumRx Select Highly Managed
OptumRx Select Managed
OptumRx Select Managed with Focused UM
Oregon Educators Benefit Board
Oregons Health CO-OP HIX
Oscar CA HIX
Oscar HIX NY
Oscar NJ HIX
Oscar TX HIX
Oshkosh Corporation/JLG Industries

© 2022 Nguyen TD et al. *JAMA Health Forum.*

PA Employees Benefit Trust Fund
PBM Plus
PacificSource HIX ID
PacificSource HIX MT
PacificSource HIX OR
Paramount Healthcare
Paramount Healthcare PPO
Paramount Individual Exchange and Essential (HIX OH)
Partners Community Healthcare
Perdue Farms
Pfizer Prescription Drug Program
Phoenix Choice Health Plan HIX
Physicians Health Plan of Michigan
Physicians Health Plan of Michigan HIX
Physicians Health Plan of Northern Indiana
Physicians Health Plan of Northern Indiana HIX
Physicians Plus Five Tier Premier
Physicians Plus Four Tier Legacy
Physicians Plus Three Tier Legacy
Physicians Plus Two Tier Legacy
Piedmont Community HealthCare HIX
Pinal County Employee Benefit Trust
Pinnacle RxSolutions Performance Drug List
PreferredOne Formulary
Premera Blue Cross Value-Based
Premera Generics Only
Premera HIX X1
Premera HIX X3
Premera HIX X4
Premera Incentive 2-Tier
Premera Incentive 4-Tier
Premera-Select
Premier Health Business Value
Premier HealthOne HIX
Presbyterian Health Plan Centennial Care
Presbyterian Health Plan FEHBP
Presbyterian Health Plan Group 4 Tier
Presbyterian Health Plan HIX NM
Prevea360 HIX WI
PrimeChoice Accord 3 Tier
Prince Manufacturing Formulary
Priority Health Michigan Commercial Formulary
ProCare Rx
Prominence Health Plan
Prominence Health Plan Nevada
Prominence Health Plan Nevada HIX
Prominence Health Plan Texas
Providence Health Plan Formulary A
Providence Health Plan Formulary D
Providence Health Plan Formulary G
Public Education Employees' Health Insurance Plan
Public Employees Health Program (PEHP)
QualChoice Basic
QualChoice Enhanced
QualChoice Essential Formulary HIX
QualChoice FEHBP
Raytheon
Regence BlueShield Essential WA HIX
Regence Commercial
Riverside Health
Rock County Formulary (Plan 2)
Rock County Formulary (Plan 4)
Rock County Formulary (Plan 6)
Rocky Mountain Health Plan
Rocky Mountain Health/ Good Health
Rural Arizona Group Health Trust
SIHO HIX
Samaritan Choice Plan
San Luis Valley HMO Colorado Two Tier
Sanford Health Plan Commercial 3 Tier
Sanford Health Plan HIX
Santa Clara County Employee
Sargento Foods
Sartori Foods
Sav-Rx
Scheurer Hospital Formulary
School District of Cudahy
Scott & White Health Plan 3 Tier with Specialty
Scott & White Health Plan 4 Tier with Specialty
Scott & White Health Plan HIX
Scott and White Health Plan ERS
ScripNet Worker's Compensation Drug List
Script Care
ScriptGuideRx/4D
Seaver Associates Leasing
Security Health Plan of Wisconsin
Security Health Plan of Wisconsin HIX
SelectHealth RxCore Idaho HIX
SelectHealth RxCore Utah
SelectHealth RxSelect Idaho
SelectHealth RxSelect Utah
Self-Insured Schools of California (SISC)
Serve You Select
Serve You Standard
Sharp Health Plan Four Tier
Sharp Health Plan Four Tier HIX
Sharp Health Plan Three Tier
Sierra Health and Life Four Tier
Sierra Health and Life Three Tier
SignatureScripts from OhioHealth Group
Southeastern Indiana Health Organization (SIHO)
State Employees of Montana Formulary
State of Arizona Employees
State of Connecticut Employee Formulary
State of Florida Employee Prescription Plan

© 2022 Nguyen TD et al. *JAMA Health Forum.*

State of Louisiana - Office of Group Benefits
State of Maryland Employees
State of Michigan Employees
State of New Jersey Employees
State of New York PICA Program
State of South Dakota Employees
State of Tennessee ParTNers for Health
State of WI Group Health
SummaCare
SummaCare HIX OH
Sutter Health
TREK Bicycle Corporation
TRS-ActiveCare
TRUSTED Health Plan
Teck American Incorporated
The Health Plan of Upper Ohio Valley
Thedacare
Total Care
Total Health Care 3 Tier
Total Health Care MI HIX
Trillium Vital Fit Standard HIX OR
Triple-S Commercial
Triple-S FEHBP
Tufts Health Direct HIX
Tufts Health Plan
Tufts Health Plan HIX
Tufts Health Plan Rhode Island
UAW Retiree Medical Benefits Trust
UCare Choice HIX MN
UPMC Your Choice For FEHBP
US Bank
Unicare State Indemnity Plan
Uniform Medical Plan
Union Pacific Railroad Employees Health System (UPREHS)
United Federation of Teachers Welfare Fund
United Healthcare California Advantage HIX
United Mine Workers of America Health & Retirement Funds
UnitedHealthCare California SignatureValue 2 Tier
UnitedHealthCare California SignatureValue 3 Tier
UnitedHealthCare Oxford Connecticut Traditional Three Tier
UnitedHealthCare Oxford Health Plan NJ/NY Traditional Three Tier
UnitedHealthCare SignatureValue 3 Tier
UnitedHealthcare 3 Tier Advantage HIX - MA
UnitedHealthcare 3 Tier Advantage HIX - NY
UnitedHealthcare 3 Tier Traditional HIX - CT
UnitedHealthcare 3 Tier Traditional HIX - NJ
UnitedHealthcare 4 Tier Advantage HIX - TN
UnitedHealthcare 4 Tier Advantage HIX AL
UnitedHealthcare 4 Tier Advantage HIX AR
UnitedHealthcare 4 Tier Advantage HIX FL
UnitedHealthcare 4 Tier Advantage HIX GA
UnitedHealthcare 4 Tier Advantage HIX LA
UnitedHealthcare 4 Tier Advantage HIX MD
UnitedHealthcare 4 Tier Advantage HIX MS
UnitedHealthcare 4 Tier Advantage HIX NC
UnitedHealthcare 4 Tier Advantage HIX RI
UnitedHealthcare 4 Tier Advantage HIX SC
UnitedHealthcare 4 Tier Essential HIX KS
UnitedHealthcare 4 Tier Essential HIX KY
UnitedHealthcare 4 Tier Essential HIX NE
UnitedHealthcare 4 Tier Essential HIX OK
UnitedHealthcare 4 Tier Essential HIX WA
UnitedHealthcare 4 Tier Essential IA
UnitedHealthcare 4 Tier Traditional HIX AR
UnitedHealthcare Commercial 3 Tier
UnitedHealthcare Essential HIX - VA
UnitedHealthcare Essential HIX AZ
UnitedHealthcare Essential HIX CO
UnitedHealthcare Essential HIX IL
UnitedHealthcare Essential HIX IN
UnitedHealthcare Essential HIX MD
UnitedHealthcare Essential HIX MI
UnitedHealthcare Essential HIX MO
UnitedHealthcare Essential HIX OH
UnitedHealthcare Essential HIX PA
UnitedHealthcare Essential HIX TX
UnitedHealthcare Essential HIX WI
UnitedHealthcare FEHBP - Choice Plus Advanced
UnitedHealthcare FEHBP - MD IPA
UnitedHealthcare FEHBP California
UnitedHealthcare FEHBP Texas
UnitedHealthcare FEHBP of the Midwest
UnitedHealthcare Golden Rule Four Tier
UnitedHealthcare Golden Rule Three Tier
UnitedHealthcare Plan River Valley - Traditional
UnitedHealthcare SignatureValue OR
UnitedHealthcare SignatureValue Open
UnitedHealthcare SignatureValue TX
UnitedHealthcare SignatureValue WA
UnitedHealthcare Traditional Four Tier
UnitedHealthcare Traditional Three Tier
UnitedHealthcare of California SignatureValue 4 Tier
Unity Health Plan Closed WI HIX
Unity Health Plan Open WI HIX
Unity Health Plan Standard Four Tier
Unity Health Plan Standard Three Tier
Univera Healthcare NY HIX
Universal Rx Focus Drug Formulary
Universal Rx Outcomes Drug Formulary
University Health Alliance 3 Tier
University Hospitals
University of Arkansas 3 Tier
University of Colorado
University of KY 3 TIER
University of Michigan Formulary

© 2022 Nguyen TD et al. *JAMA Health Forum.*

University of Minnesota UPlan Formulary
University of Toledo
University of Utah HIX
VIVA Essential Health Benefit Formulary
VIVA Essential Health Benefit Formulary with Value Generics
VIVA Health Advanced Formulary
VIVA Health Advanced Formulary with Value Generics
VRx Commercial Formulary
Valley Health Plan HIX CA
Vanderbilt University
Vantage Health Plan LA
Ventegra Preferred Formulary
Ventegra Premium Formulary
Ventegra Premium Plus Formulary
Ventura County Health Care Plan
Virginia Premier
Virginia Premier Elite
Viva Health Alabama
Viva Health UAB
Walmart Associates Formulary
Walmart Generics Program
WellCare of Kentucky HIX
WellCare of New York HIX
WellDyneRx Formulary
Wellmark Blue Rx Preferred 3 Tier with Specialty
Wellmark Blue Rx Preferred 4 Tier with Specialty
West Virginia PEIA
Western Health Advantage Commercial
Western Health Advantage HIX CA
Wexford Health Corporate Formulary
Whirlpool Corporation
Wisconsin Diagnostic Laboratories
Wisconsin Physician Services
Yale University Health Plan
Zoom Health OR HIX

© 2022 Nguyen TD et al. *JAMA Health Forum.*
